# Secondary Hemorrhages

**Published:** 1877-03

**Authors:** A. D. L. Napier


SECONDARY HEMORRHAGES.—*By A. D. L. Napier, M.B., C.M., etc.*—Hemorrhage is, and always must be, one of the most important subjects which the obstetrician has to consider. It is one of the most dangerous consequences attending parturition; yet, fortunately, with clearly arranged ideas, and the prompt execution of remedial measures, our treatment is usually attended with speedy and marked success. Secondary hemorrhages may be divided into two great classes—(1) dependent on the muscular portion of the uterus; (2) dependent on the vascular uterine system.

(1.) This class is not generally recognized as secondary hemorrhage, probably because it frequently escapes attention. The physiological action of the uterine muscular layers, subsequent to labor, has been long admitted; if the contraction is insufficient, what is known as sub-involution is the consequence.

Sub-involution may occur (*a*) after prolonged and difficult labors; (*b*) after very rapid and easy labors in women of relaxed tissues, more especially in multiparæ; (*c*) in consequence of uterine disease, tumors, etc.; (*d*) in consequence of too early resumption of the erect posture; (*e*) from retained secundines. In short, sub-involution may arise from any source which causes insufficient uterine contraction.

As a general rule this affection is sub-acute, the discharge being most frequently a prolongation of the colored lochial flow; in these cases, absolute rest, and the exhibition of ergot, gallic acid, or iron is usually all that is required. A case, which proved an exception to this rule, came under my treatment in January, 1876. The subject was an unmarried girl, servant to a gentleman residing about eight miles from Fraserburgh; she informed me that she had been "quite regular," but that her "present illness had lasted three weeks." I ordered the usual treatment, and did not see her again for several days, when she was a little better, and was ordered absolute rest in bed. I saw her again on 28th January (six days from my last visit), and finding her no better, insisted on making a thorough examination. The os was soft and open; the uterus measured longitudinally 4½ inches. On the night of 29th January I was sent for. On my arrival I found she had been up the previous day after my visit, and that the discharge had been considerably increased; notwithstanding she again rose on the evening of the 29th, when a great gush of blood came on, and had been flowing incessantly since. The patient was quite blanched, insensible, and almost pulseless. Losing no time, I cleared the vagina, and the os being patulous, injected a solution of the perchloride of iron, which eventually arrested the bleeding; a vaginal tampon of cotton wool, saturated with glycerine and liquor ferri perchlor. was applied, and a full dose of ergot ordered every three hours; pads were firmly fixed over the uterus. In an hour or two she was much better, but I did not feel justified in leaving that night. Next day she was much improved; the vaginal plug was removed; a vaginal injection of perchloride of iron and water was ordered to be given twice or thrice daily if the hemorrhage should be excessive. Visited again on 1st February, when the discharge was in very moderate quantity. On the 6th February I found that, to make matters sure, the iron injections had been duly continued, although the bleeding was stopped. By 8th February she was quite recovered, and the uterus restored to almost its normal dimensions. It is right to add that in this case I was fully persuaded that the girl had aborted, though her vehement denial at first staggered me.

Secondary hemorrhage proper is distinguished from sub-involution by this simple fact; in the former the lochial flow is not prolonged beyond usual, but without any warning a sudden and excessive hemorrhage occurs. This is owing to the condition of the uterine vessels, and may result from (1) imperfect thrombosis of a uterine vessel, which may be owing to (*a*) a deficient power of defibrination in the blood; (*b*) too early retraction of the vessel, by which, so to speak, the vessel is pulled away from its plug; (2) imperfect, or rather irregular, uterine contraction, giving rise to insufficient thrombosis. This is distinguished from that formerly described thus: In sub-involution the inertia is general, in secondary hemorrhage partial, so that for a time the pressure from the surrounding contracted tissues is adequate to sustain the closure of the sinus; but eventually, when the other vessels are sufficiently occluded, and the uterus begins to lose tonicity, the relaxed tissues become insufficient, the imperfect thrombus is detached, and bleeding begins. Again, under this variety we have the formation and subsequent detachment of polypoid tumors; bleeding on account of uterine malposition which, after the process of involution, become rectified in part only, etc.

It may be objected that I would establish a distinction, without a real difference, but without such distinction it seems impossible to realize the import of some cases.

As cases of secondary hemorrhage are comparatively rarely seen, I may be excused for giving the following:

Ann C., residing in the village of Broadsea, unmarried, was delivered by Mrs. S., midwife, on 2d November, 1876. Lobor was natural, and from the midwife's experience, I have every reason to believe she was properly treated. The sanguineous discharge ceased on 7th November, the fifth day; on the 9th November she rose, had her bed made, and felt very well; 10th November rose at 11 A.M., sat by the fire; she did not exert herself in any way; the only thing she did at all accounting for the after symptoms was, to use her mother's words, "took a pull at the cradle." About 7 P.M., while sitting by the fire, a frightful gush of blood suddenly came from her, and she lost an enormous quantity before she could be removed to bed. The midwife who attended her was immediately sent for, and did what she could to arrest the bleeding, but failed to do so satisfactorily. About nine o'clock I was desired to see her, and at once visited.

On my arrival the condition of the girl was: Face quite blanched, perfectly colorless, eyes sunken, mouth slightly open, skin of body cold and clammy; the pulse could hardly be felt at the wrist, and beat over 120 a minute; respiration was very slow and sighing. I took a hurried glance at the clothes which had been used to protect the bed, and it is no exaggeration to write that four or five large flannel petticoats, besides as many other pieces of cloth of like size, were perfectly saturated; the bed was completely soaked. The quantity of blood lost must have been immense. On making an external examination, I found the uterus floating in the lower part of the abdomen, and thereon made a vaginal exploration. The lower part of the canal was filled with clotted blood, which I removed in great quantity: the posterior cul-de-sac also contained a number of clots, so that the vagina was almost full, yet blood continued to flow freely. Having emptied the vagina, after soaking both my hands in very cold water, I proceeded to explore the uterus; one hand fixed the organ externally, and the other was applied internally. I found the os sufficiently open to admit two fingers, and removed some clots from the lower part of the womb. I then had the satisfaction of feeling the organ contract under my hand. After taking away as much clotted blood as I could, I broke up all I could reach, and by this time the hemorrhage was controlled. I prescribed ergot in the fluid form, combined with liquor strychniæ and liquor morphiæ, and ordered cloths steeped in cold water to be applied over the uterus and vulva, to be renewed every ten or fifteen minutes for an hour and a half; thereafter the binder to be applied; also that half an ounce of brandy every two hours and beef tea *ad lib.*. were to be given. 11th Nov.—A small quantity of red discharge, no return of hemorrhage; the patient's condition was wonderfully improved. She was ordered a vaginal douche of cold water, and to continue her medicine. 12th Nov.—I was informed that some clotted blood came away on the afternoon of the previous day; uterine contraction was good. By the 14th the discharge had ceased, and she was able to renew the nursing of her baby. The above is a typical case of secondary hemorrhage, occurring as it did on the eighth day, three days after the colored lochia had disappeared. "Of twenty-five cases recorded by Drs. M'Clintock and Hardy, only one occurred so late as the seventh day" (Churchill, p. 487).

Briefly to resume our consideration of these hemorrhages. It is evident that as they are different in themselves, so must we consider them essentially different in their causation. I have adverted to the retention of part of the secundines as a cause of sub-involution, but I wish to qualify the assertion. Secundines, unless in large quantity, are not apt to cause sub-involution; and if in large quantity, other results are more likely to accrue. If in small quantity, especially in certain conditions of the uterine lining, they will more probably give rise to irregular contraction, and hence to secondary bleeding. Again, in sub-involution we see simply an impairment of a normal condition; in secondary hemorrhage, purely an abnormal phenomenon, probably resulting from a special dyscrasia. Mental emotion may in some measure tend to the production of the latter. It is allowed (Dr. Ayre) that liver disease has brought about secondary hemorrhage, nervous influences acting on the heart and general blood supply may likewise do so. Be this as it may, I imagine that in the foregoing case mental distress was at least partly to blame. I found, on inquiry, that my patient had been seduced, and that the man had not fulfilled his promise of marriage, in consequence of which she had been greatly distressed.

In distinguishing the two, the best clinical distinction is the state of the os, but the size of the uterus and the previous history will also guide us.

*Treatment.*—I have already indicated that in most cases of sub-involution, medicinal treatment, rest, and properly applied uterine pressure are sufficient. We should have no fear of using iron injections in such cases, because the os is patulous, and the return of the injected fluid almost certain. The absorptive function is not active, which fact was demonstrated in the first case cited, so that we have nothing to fear from pyæmia. In secondary hemorrhage medicines may be of use to a certain extent, but we have no time for their action. At first we must cause contraction, which will be best done manually. Galvanism has been shown to be uncertain as a stimulator to uterine contraction; besides, we have not always a battery at hand; the intra-uterine injection of perchloride of iron would be dangerous with a contracted os, and we cannot spare time to dilate it; if we feel clots which the finger cannot reach properly, they might be carefully broken by some instrument introduced into the uterus. A cold water vaginal douche may be of service, as it undoubtedly frequently causes contraction; plugging the vagina is worse than useless, unless we can exert sufficient pressure on the uterus from above. I do not here consider the special treatment applicable to these secondary hemorrhages which arise from special causes, such as rupture of a vein at the cervix, ulceration of the cervix, or rupture of a varicose vulvular vein. Of internal treatment, brandy, with beef tea, and ergot, must stand foremost; and in connection with ergot, I must utter my conviction of the greatly increased specific action of this drug if we add strychnia to it. This, if I mistake not, has been advocated by the Dublin school, notably by Dr. Athill, and deserves due consideration. To sum up. In sub-involution, all other things failing, try to form artificial thrombi; in secondary hemorrhage, let us first gain uterine contraction.—*Obstetrical Journal.*

